# Kampo herbal ointments for skin wound healing

**DOI:** 10.3389/fphar.2023.1116260

**Published:** 2023-02-10

**Authors:** Manon Paul-Traversaz, Kaoru Umehara, Kenji Watanabe, Walid Rachidi, Michel Sève, Florence Souard

**Affiliations:** ^1^ Univ. Grenoble Alpes, CNRS, TIMC UMR 5525, EPSP, Grenoble, France; ^2^ Yokohama University of Pharmacy, Kampo Natural Product Chemistry Laboratory, Yokohama, Japan; ^3^ Yokohama University of Pharmacy, Yokohama, Japan; ^4^ Univ. Grenoble Alpes, CEA, Inserm, IRIG-BGE, Grenoble, France; ^5^ Univ. Grenoble Alpes, CNRS, DPM UMR 5063, Grenoble, France; ^6^ Univ. libre de Bruxelles, Department of Pharmacotherapy and Pharmaceutics, Faculty of Pharmacy, Brussels, Belgium

**Keywords:** Kampo, ethnopharmacology, skin, wound healing, traditional medicines, herbal medicines

## Abstract

The management of skin wound healing problems is a public health issue in which traditional herbal medicines could play a determining role. Kampo medicine, with three traditionally used ointments, provides interesting solutions for these dermatological issues. These ointments named *Shiunkō*, *Chuōkō*, and *Shinsen taitsukō* all have in common a lipophilic base of sesame oil and beeswax from which herbal crude drugs are extracted according to several possible manufacturing protocols. This review article brings together existing data on metabolites involved in the complex wound healing process. Among them are representatives of the botanical genera *Angelica*, *Lithospermum*, *Curcuma*, *Phellodendron*, *Paeonia*, *Rheum*, *Rehmannia*, *Scrophularia*, or *Cinnamomum*. Kampo provides numerous metabolites of interest, whose content in crude drugs is very sensitive to different biotic and abiotic factors and to the different extraction protocols used for these ointments. If Kampo medicine is known for its singular standardization, ointments are not well known, and research on these lipophilic formulas has not been developed due to the analytical difficulties encountered in biological and metabolomic analysis. Further research considering the complexities of these unique herbal ointments could contribute to a rationalization of Kampo’s therapeutic uses for wound healing.

## 1 Introduction

Eight million people suffer from chronic wounds worldwide, and the annual wound care products’ market is expected to reach $15–22 billion by 2024 (Sen, 2019; Bowers and Franco, 2020). These dermatological lesions have a significant impact on public health and have psychological consequences (Tzaneva et al., 2016). The ineffectiveness of topical treatments derived from synthetic chemistry, the occurrence of antibiotic resistance, and the predominant use of surgery (mostly debridement methods) to treat skin ulcers challenged the therapeutic solutions that could be offered to patients suffering from chronic wounds or severe burns (Elraiyah et al., 2016).

In this publication, we review the solutions brought by the Japanese traditional medicine, named Kampo (漢方医学). Kampo is largely inspired by traditional Chinese medicines (TCMs) since its history begins in Japan in the sixth century when it was imported from China. Over the centuries and with practice, it has become more specific and different from Chinese medicines, having its own identity. Kampo uses its own diagnostic techniques and therapeutics where plants have a predominant role. The remedies involve several crude drugs, such as parts of plants, mushrooms, animals, and minerals, combined in remedies named formulas (カンポ式). These formulas are numerous and prescribed according to the uniqueness of the patient and their symptoms. Thus, Kampo is considered a holistic and integrative medicine. The traditional approach and unique compositions of herbal crude drugs encourage interest in research in areas where modern medicine may be insufficient in terms of therapeutic solutions offered to patients. There are several hundred Kampo formulas and variations. Out of them, 148 formulas are approved as prescription drugs and are covered by the Japanese health insurance system (Watanabe et al., 2011). Among these traditional therapeutics, there are three formulas for topical use: *Shiunkō* (しうんこう; 紫雲膏)*, Chuōkō* (ちゅうおうこう; 中黄膏), and *Shinsen taitsukō* (しんせんたいつこう; 神仙太乙膏). They are traditionally been used on wound healing processes ranging from hemostasis and inflammation to proliferation and remodeling phases (Lu et al., 2008; Chak et al., 2013).

Wound healing is a natural physiological response to tissue damage. This complex biological process implicates different cell types and chemical interactions involving cytokines and other mediators. Experimental works have revealed the general steps in the wound healing process. This complex process is organized into four sequential steps. Hemostasis is the first stage, triggered by the body to stop the excessive loss of blood. Then, the inflammatory phase mediated by macrophages and growing factors is also initiated to fight any possible infection. Once the wound is stable, the proliferation phase, which is longer and can last several weeks, starts and comprises granulation, re-epithelialization, and neovascularization. To finish, there is the maturation and remodeling phase (Cañedo-Dorantes and Cañedo-Ayala, 2019; Wallace et al., 2022). In chronic injuries, these mechanisms are impaired. This category of lesions including vascular ulcers, arterial ulcers, pressure ulcers, and chronic ulcerations in diabetic mellitus has common causal factors such as aging, hypoxia, or bacterial colonization. These factors will influence the inflammatory step with modifications to the number of cells involved (more neutrophils and macrophages) and the mediators and cytokines secreted. Chemical modulations include an increase in pro-inflammatory cytokines, metalloproteases, reactive oxygen species (ROS) and a decrease in growth factor availability. All of these unfavorable parameters lead to cell death and destruction of the extracellular matrix (Zhao et al., 2016). Indeed, for chronic diabetic wounds, some disturbances in angiogenesis, neovascularization, cell function or failure in matrix metalloproteinases (MMPs), and keratinocyte and fibroblast functions may affect wound healing mechanisms a lot (Ghaisas et al., 2014).

The use of topical products from the Kampo traditional medicine may play a beneficial role in the therapeutic management of chronic wounds and in the wound healing process. However, these remedies have so far been little studied. If Kampo medicine is recognized for its innovative standardization with regards to a large number of drinkable extracts, this is not yet the case for therapeutic forms related to dermatological uses. Therefore, research on Kampo ointments could lead to a better understanding of the metabolites and therapeutic mechanisms involved.

## 2 Materials and Methods

We reviewed the wound-healing Kampo ointments, the oily excipients and crude drugs used, and their wound-healing therapeutic activities related to specific metabolites. We consulted the PubMed database, and the keywords favored for the bibliographic searches were the following: Kampo, wound healing, skin, chronic wounds, ulcers, diabetic foot ulcers, burns, keratinocytes, fibroblasts, endothelial cells, epithelialization, common or Latin names of the plants included, and major metabolites involved in the wound healing process.

Moreover, information about crude drugs from Kampo-related databases such as the Center for Kampo Medicine, Keio University School of Medicine, and the KampoDB, along with metabolites data, enhanced our research work (Sawada et al., 2018; Keio University Kampo Department). All the metabolites mentioned are important for future research studies based on metabolomic analyses. They are presented with their molecular formula and exact mass with four digits after the decimal point (Kim et al., 2023).

## 3 Wound-healing herbal topical therapies from Kampo

### 3.1 Introduction about the Kampo ointments

There are mainly two topical preparations used in Kampo medicine to treat chronic wounds and burns. A third, coming from TCM, can be added to the list. Both Japanese formulas were created during the Edo period (1603–1867) by the medical doctor Hanaoka Seishū (1760–1835). These two remedies are named *Shiunkō* and *Chuōkō*. Each of the two formulas includes two herbal crude drugs extracted into a lipophilic base composed of sesame seed oil. Both contain beeswax but *Shiunkō* has the specificity of a lard addition. Beeswax is added before or after the herb extraction step according to manufacturing protocols. The third topical formula is called *Shinsen taitsukō*. This one is characterized by a larger number of herbal ingredients and the absence of lard. All crude drugs are described in Table 1 (The society of Japanese pharmacopeia, 2016). These three multiherbal topical preparations are used by Kampo health professionals to treat dermatological disorders. The application fields are similar for all formulas that are dedicated to wounds and burns care, thanks to a wound healing action. These therapeutic indications are detailed in Table 2.

**TABLE 1:** Herbal medicines from Kampo topical ointments: Japanese and Latin denominations, plant organ used, and botanical family for each plant.

Kampo ointments	Herbs	Organ	Botanical family	Japanese names	
*Shiunkō* 紫雲膏 しうんこう	*Angelica acutiloba* (Siebold & Zucc.) Kitag. *Yamato-tōki* (ヤマトトウキ) or *Angelica acutiloba* var*. Sugiyamae* Hikino *Hokkai-tōki* (ホッカイトウキ)	Root	*Apiaceae*	トウキ*tōki*	当 帰
	*Lithospermum erythrorhizon* Siebold et Zucc.		*Boraginaceae*	シコン *shikon*	柴
					根
*Chuōkō*中黄膏ちゅうおうこう	*Curcuma longa* L.	Rhizome	*Zingiberaceae*	ウコン*ukon*	鬱 金
	*Phellodendron amurense* Rupr. or *P. chinense* C.K.Schneid	Bark	*Rutaceae*	オウバク *ōbaku*	黄 柏
*Shinsen taitsukō* 神仙太乙膏 しんせん たいつこう	*Angelica acutiloba* (Siebold & Zucc.) Kitag.	Root	*Apiaceae*	トウキ	当
				*tōki*	帰
	*Angelica dahurica* (Hoffm.) Benth. & Hook.f. ex Franch. & Sav.	Root	*Apiaceae*	ビャクシ *byakushi*	白 芷
	*Cinnamomum cassia* (L.) J. Presl (synonym: *Cinnamomum cassia* Nees ex Blume)	Bark	*Lauraceae*	ケイヒ	桂
				*keihi*	皮
	*Paeonia lactiflora* Pall.	Root	*Paeoniaceae*	シャクヤク *shakuyaku*	芍
					薬
	*Rehmannia glutinosa* (Gaertn.) DC. (synonym: *R. glutinosa* var. *Purpurea* Makino also known as *R. glutinosa f. Purpurea* Matsuda)	Root	*Orobanchaceae*	ジオウ	地
				*jiō*	黄
	*Rheum palmatum* L., *R. tanguticum* Maxim., *R. officinale* Baill., and *R. coreanum* Nakai or their interspecific hybrids	Root	*Polygonaceae*	ダイオウ	大
				*daiō*	黃
	*Scrophularia ningpoensis* Hemsl. or *S. buergeriana* Miq.	Root	*Scrophulariaceae*	ゲンジン *genjin*	玄 参

**TABLE 2:** Kampo ointments and their therapeutic indications regarding dermatology.

	Traditional therapeutic indications
*Shiunkō*	Wounds, burns, hemorrhoidal lesions, eczema and dermatitis, cracks, and chilblains
*Chuōkō*	Surgical wounds, sprain, and cutaneous infections such as furuncles
*Shinsen Taitsukō*	Wounds, burns, insect bites, itching, and pressure ulcers

These ointments have singular, bright colors: purplish-red for *Shiunkō*, bright yellow for *Chuōkō*, and greenish-yellow for *Shinsen taitsukō.* This color aspect is important from an ethnopharmacological point of view because Kampo health professionals attribute it to potent antioxidant beneficial actions. To our knowledge, these considerations for Kampo ointments have not been validated while using analytical methods.

The *Shiunkō* formula contains roots from purple gromwell (*Lithospermum erythrorhizon* Siebold & Zucc.; Boraginaceae) and Japanese angelica (*Angelica acutiloba* Siebold & Zucc. Kitag.*/A. acutiloba* Kitag. var*. sugiyamae*; Apiaceae). Regarding the relative concentration of herbal crude drugs, purple gromwell root constitutes 75% of the herbal mixture and 25% of Japanese angelica root (Japan Pharmaceutical Manufacturers Association, 2005). *Shiunkō* is a standardized formula that has been covered by National Health Insurance (NHI) since June 1984 and is widely produced by Tsumura & Co., leader of the production of Kampo health products in Japan. Like all Kampo preparations produced by Tsumura, *Shiunkō* has its own identification number (T501) (Tsumura & Co, 2017). The formula is distinguished from *Chuōkō* by the presence of an additional ingredient, which is lard. It is used in small amounts (1.3%) and in some extraction protocols only. *Shiunkō* is traditionally involved in the treatment of wounds, burns, hemorrhoidal lesions, eczema, dermatitis, cracks, and chilblains (Fukurodo Pharmacy).

*Chuōkō* formula contains a mixture of turmeric (*Curcuma longa* L.; Zingiberaceae) and Amur cork tree bark (*Phellodendron amurense* Rupr. or *P. chinense* C.K.Schneid.; Rutaceae). The relative concentration of herbal crude drugs in the ointment is 75% for turmeric and 25% for Amur cork tree bark, extracted in similar lipophilic extraction conditions as for *Shiunkō. Chuōkō* is used to care for surgical wounds, sprains, and cutaneous infections such as furuncles (Fukurodo Pharmacy).

The *Shinsen Taitsukō* formula is the most complex with seven crude drugs mixed in equal quantities. It includes roots from Japanese angelica (*Angelica acutiloba* (Siebold & Zucc.) Kitag.)*,* Dahurian angelica (*Angelica dahurica* (Hoffm.) Benth. & Hook. f. ex Franch. & Sav*.*), Chinese peony (*Paeonia lactiflora* Pall.), Chinese rhubarb (*Rheum palmatum* L., *R. tanguticum* Maxim., *R. officinale* Baill., and *R. coreanum* Nakai or their interspecific hybrids.), figwort (*Scrophularia ningpoensis* Hemsl. or *S. buergeriana* Miq.), Chinese foxgloves (*Rehmannia glutinosa* (Gaertn.) DC.), and Chinese cinnamon bark (*Cinnamomum cassia* (L.) J. Presl). All these herbs are listed in the Japanese pharmacopoeia except the two species from the *Scrophularia* genus, which are more connected to TCM and related to the *Shinsen Taitsukō* Chinese origin. *Shinsen Taitsukō* is traditionally used in the care of wounds, burns, insect bites, itching, and pressure ulcers (Fukurodo Pharmacy).

About herbal ingredients and their taxonomic aspects (Table 1), two main inconsistencies are raised between the Latin names mentioned in the 17th edition of the Japanese Pharmacopoeia and those recognized as being up-to-date by World Flora Online (WFO), a project of the United Nations Convention on Biological Diversity (WFO, 2022). First, taxonomic ambiguity concerns *Angelica acutiloba* and the variety *A. acutiloba* var. *sugiyamae*, presented as distinct in the Japanese Pharmacopeia (The society of Japanese pharmacopeia, 2016). The second ambiguity is related to *Rehmannia glutinosa* (Gaertn.) DC. and its variety, *R. glutinosa* var*. purpurea* Makino (now named *Rehmannia glutinosa f. purpurea* Matsuda) (WFO, 2022). Indeed, the Japanese Pharmacopeia prefers to consider the old nomenclature while distinguishing *R. glutinosa* Liboschitz var. p*urpurea* Makino from *R. glutinosa* Liboschitz (The society of Japanese pharmacopeia, 2016). These taxonomic ambiguities add further complexity to the collection of accurate information about crude drugs’ botanical origin. In addition, for the more traditional and artisanal ointments manufacturing processes, there are important variations in the use of botanical crude drugs. Examples are the possible replacement of *Lithospermum erythrorhizon* root by *Arnebia euchroma* root or the fact that figwort roots can be derived indifferently from *Scrophularia ningpoensis* or *S. buergeriana*. These specificities are not mentioned in the Japanese Pharmacopoeia, which underlines the necessity of developing more exhaustive monographs in the future.

### 3.2 Extraction methods for Kampo topical remedies

**FIGURE 1:**
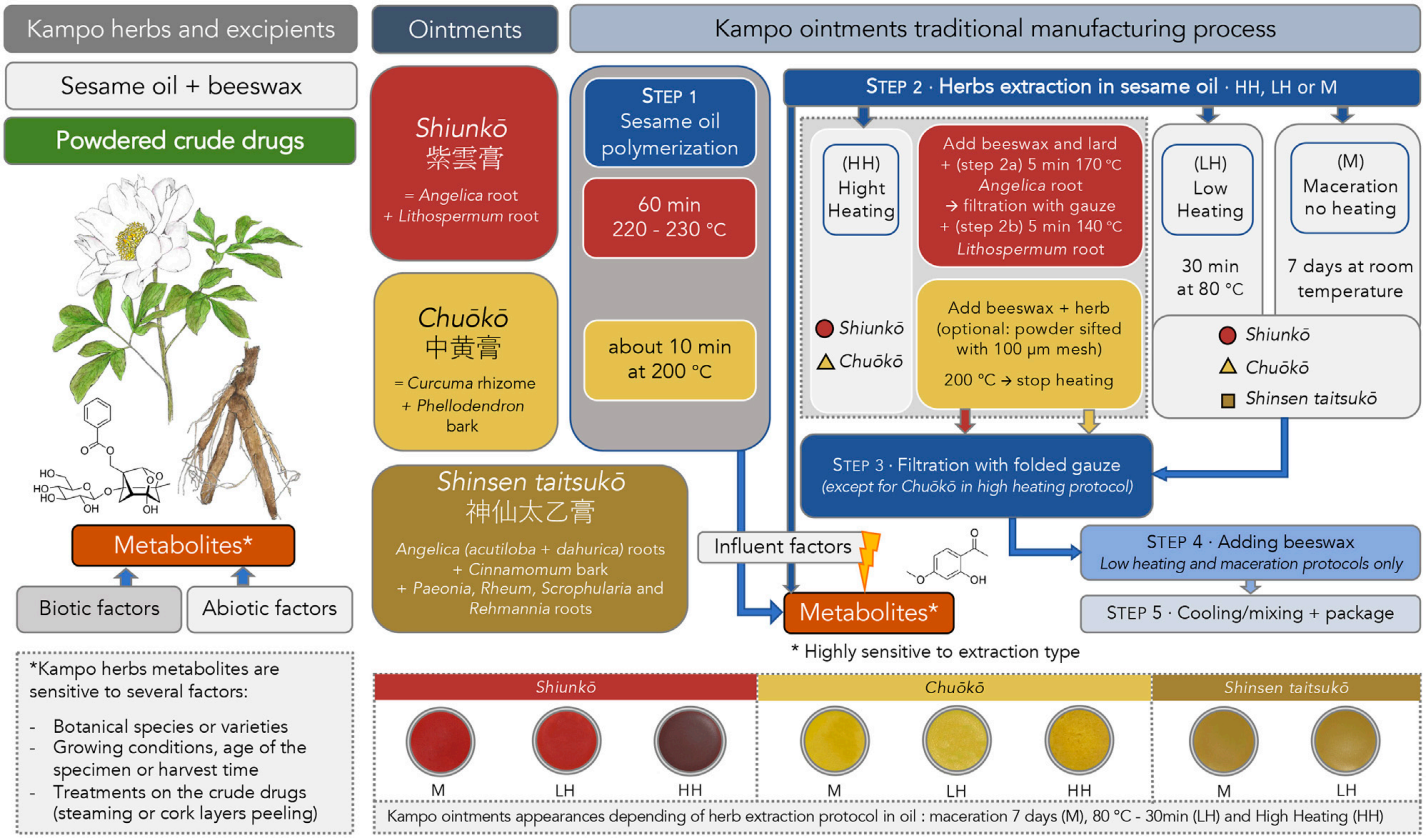
Descriptive diagram presenting the traditional manufacturing process for Kampo ointments (*Shiunkō, Chuōkō,* and *Shinsen taitsukō*) and its implications on herb metabolites and ointments appearances.

The making process of these three Kampo ointments is, for the most part, traditional and devoid of consensual information regarding dosages and extraction protocols. Preparation processes dating from the Edo period (1603–1867) do not mention any precise timing or temperature of extraction in sesame oil. Nevertheless, organizations related to the Japanese Pharmacopoeia offer handbooks that can serve as a reference for these making protocols. Some official recommendations reference the manufacturing processes and the quantities of herbal drugs needed (Table 3) (Japan Pharmaceutical Manufacturers Association, 2005). All information regarding the manufacturing process is summarized in Figure 1.

**TABLE 3:** Ingredients in Kampo ointments and corresponding quantities and fractions for excipients and herbs.

	Quantities of ingredients (g)					
	*Shiunkō*		*Chuōkō*		*Shinsen Taitsukō*	
Sesame oil	1,000		1,000		1,000	
Beeswax	340		380		400	
Lard	20		–		–	
Herb a	*L. erythrorhizon*	120	*C. longa*	40	–	
Herb b	*A. acutiloba*	60	*Phellodendron sp.*	20	–	
Mix of herbs	–		–		*A. acutiloba, A. dahurica,* and *C. cassia,*	32
					*P. lactiflora, R. glutinosa, Rheum sp.,* and *Scrophularia sp.*	
	Fractions (%)					
Percentage of excipients	88.3		95.8		86.2	
Percentage of herbs	11.7		4.2		13.8	

For *Shiunkō* preparation, the most common making protocol (HH) consists of successive 5 min extractions in sesame oil at 170°C (step 2a) for the Japanese angelica root and then at 140°C for the *Lithospermum* root (step 2b). For 1,000 g of sesame oil, 120 g of *Lithospermum* root and 60 g of Japanese angelica root are used. Extraction is carried out in previously obtained polymerized sesame oil at high temperature (220°C–230°C) for one hour (step 1). Beeswax and lard are added before the extraction process. After extraction, crude drugs are filtered (step 3). Cotton gauze pads are used for filtration. According to the protocols, melted beeswax is added after extraction and filtration (step 4). During the cooling process, the mixture is homogenized on a glass plate and then packaged (step 5).

Regarding *Chuōkō*, recommendations advise a hot temperature extraction protocol (HH) with polymerized oil as well but for a shorter period (about 10 min at 200°C) (step 1). Extraction is carried out in a mixture of sesame oil and beeswax heated to 200°C until cooling (step 2). Powdered herbs could be sifted (using a 100-µm mesh) first, and then there is no filtration after extraction and no lard added. This optional filtration step is a factor of variability in the final composition. Indeed, the larger particles of powdered crude drugs, such as the cortex of *C. long*a or fibers from *Phellodendron sp.*, would be less represented in the final product. To finish, the mixture is cooled, homogenized on a glass plate, and packaged (steps 4–5). For 1,000 g of sesame oil, 40 g of turmeric and 20 g of *Phellodendron* bark are used.

For the *Shinsen taitsukō* formula, there is no fixed established protocol. Only the quantities are mentioned (Japan Pharmaceutical Manufacturers Association, 2013). Protocols use lower temperatures (step 2): 30 min at 80°C (LH) or room temperature for 7 days of maceration (M) followed by filtration (step 3) and the addition of beeswax. Mixture is then cooled and homogenized on a glass plate before packing (step 4–5). By extension, this lower extraction temperature protocol can be applied to *Shiunkō* and *Chuōkō* formulas (LH and M).

While the Japanese pharmaceutical industry favors high-temperature extractions that respect traditional recipes as they were conceived in the Edo period, the gentler ways of extraction using one week of room temperature oil maceration (M) or extraction at 80°C for 30 min (LH) are also used. These making protocols at milder temperatures are recommended and studied by some health professionals’ members from the Nihon Kampō Kyokai, 日本漢方協会, a Japanese association created in 1970 which contributes to the improvement and promotion of Kampo from the manufacturing process, distribution and dispensing of Kampo medicines (Nihon Kampō Kyokai).

Considering these different manufacturing methods, where the extraction temperature varies from room temperature to 170°C, we could imagine that the composition must certainly be different. These contrasting parameters such as the extraction temperatures or polymerization step will affect the ointment’s appearance. Indeed, for the *Shiunkō* formula, the higher the temperature, the darker the color of the ointment (Figure 1). These contrasts in color and consistency are surely correlated to differences regarding the chemistry of pigments or polyphenols. Little is known about the natural compounds extracted by those traditional processes using sesame oil. Since the several extraction methods lead to differences in the chemical composition of the ointments, the question arises as to how to control the quality of the Kampo ointments and how to regulate the manufacturing protocols. However, no proper standardization can be envisaged at the present time in view of the traditional and multi-faceted nature of the Kampo ointments manufacturing processes.

### 3.3 Lipophilic products from Kampo ointments

In Kampo ointments, there are two categories of compounds that could be related to a wound-healing action: lipophilic ones including fatty acids or ceramides and metabolites extracted from herbal crude drugs. Table 3 shows the weights and percentage ratios for the composition of Kampo ointments, with an average mass ratio close to 90% for the fatty excipients.

#### 3.3.1 Sesame oil

Fatty acids are associated with strong anti-inflammatory and antioxidant effects (Lin et al., 2017). Sesame oil obtained from *Sesamum indicum* L. (Pedaliaceae) seeds contains 45% of linoleic acid, 40% of oleic acid, 10% of palmitic acid, and 5% of stearic acid (Moore et al., 2020).

Linoleic acid 18:2 (ω-6) is a common polyunsaturated fatty acid present in plant oils. It is considered as an essential omega-6 fatty acid, a precursor of arachidonic acid 20:4 (*n*–6), prostaglandins, and leukotrienes well implicated in the inflammation process (Moore et al., 2020).

Oleic acid is a monounsaturated fatty acid called omega-9. It can be used to enhance the topical delivery of drugs and improve the trans-epithelial distribution of compounds present in the mixtures. Oleic acid could, however, cause some form of toxicity to the skin cells. This has been studied on keratinocytes even at low concentrations. In contrast, when oleic acid was applied to reconstructed human epidermis or injured skins, no alteration in morphology was observed. This is related to the modulation of the thickness of the stratum corneum, the outermost layer of the epidermis, in the control of oleic acid-induced toxicity. Low amounts of oleic acid are sufficient to cause modulation of local cytokines production without affecting skin morphology while inducing skin irritation *in vivo* (Boelsma et al., 1996). In addition, palmitic and stearic acids also have effects on the wound healing process since they can modulate cell migration, proliferation, and the production of inflammatory mediators (Silva et al., 2018).

To conclude about fatty acids content, oils with high content of linoleic acid 18:2 (*n*−6) (such as sunflower oil, *Helianthus* sp*.*, and sesame oil) are more favorable for treating skin disorders than those with high content of oleic acid 18:1 (*n*−9) (such as olive oil, *Olea europaea*) (Moore et al., 2020). Due to its important presence in Kampo ointments, sesame oil plays a decisive role. It influences the extraction of herbs and has a therapeutic emollient role.

#### 3.3.2 Oil polymerization and influences on herbs extraction

Several physicochemical steps will affect the molecular structure of sesame oil due to exposure to high temperatures (at 200°C for 10 min for *Chuōkō* to 1 h for *Shiunkō* at 220°C–230°C). One of the main transformations is the polymerization step mentioned in the Japanese reference books for Kampo formulas (Japan Pharmaceutical Manufacturers Association, 2005).

The polymerization process results in the formation of non-volatile polar compounds, triacylglycerol dimers, and polymers. Also, hydrolysis and oxidation influence the properties of the oil (Choe and Min, 2007). Dimerization and polymerization in heated oils are radical reactions that produce dimers and polymers, respectively (Khor et al., 2019). The smoke point reflects the breakdown of fats into glycerol and free fatty acids (Ramroudi et al., 2022). It depends on the free fatty acids content and the refining process used. The smoke point of sesame oil is between 184°C and 242°C, depending on the study (Ramroudi et al., 2022). The more unsaturated the vegetable oil is, the more carbon–carbon bonds it contains, and the quicker the oil will polymerize.

Thus, the polymerization of oleic acid leads to the production of acyclic polymers which will probably influence the extraction of metabolites from herbs. Linoleic acid is more easily polymerized at high temperatures than oleic acid (Choe and Min, 2007). As a result, sesame oil is subject to these changes. The interesting polymerization process, by changing the polarity of the oil, will probably influence the extraction of the crude drugs.

#### 3.3.3 Fatty acid esters and long-chain alcohols from beeswax

Beeswax has been used since ancient times for its antimicrobial properties in European and Asian traditional medicines (Cornara et al., 2017). It includes a mixture of more than 300 components such as hydrocarbons (chain length of 27–33 carbons), free fatty acids, fatty acid alcohols and esters, and exogenous substances such as propolis or pollen.

Several studies carried out on the hive products proved an antibacterial effect with an important intensity for propolis and a lighter intensity for beeswax although significant (Fratini et al., 2016). Beeswax showed *in vitro* antimicrobic effectiveness against some infectious germs tested with zone of the inhibition assay using the agar diffusion method. Among the germs tested favorably in the higher zones of inhibition, we find *Candida albicans, Staphylococcus aureus*, and *Bacillus subtilis* (Ghanem and Arabia, 2011). Also, the extracts of beeswax in methanol or ethanol have been evaluated using the zone of inhibition of the development of microorganisms. The results showed a significant inhibition zone for the beeswax methanolic extract on *S. enterica* and *C. tropicalis*. For 70% methanol extract, the most sensitive germs were *E. coli, C. albican*, *C. tropicalis*, and *Aspergillus niger* (Kacániová et al., 2012). Research works hold *in vitro* also demonstrated that the inhibitory effect is enhanced synergistically with other natural products such as propolis or olive oil (Fratini et al., 2016). Therefore, it can be assumed that it would be the same with sesame oil and herbs used for Kampo ointments. Concerning the mechanism of action and specific properties of beeswax, its antibacterial mode of action is still however poorly studied (Fratini et al., 2016). Moreover, beeswax is a difficult to standardize product and very sensitive to biotic and abiotic factors during its production by bees.

Topical application of preparations containing beeswax forms a protective barrier against several external factors by forming a film on the skin surface. Main compounds such as squalene (C_30_H_50_; 410.3912), 10-hydroxy-*trans*-2-decenoic acid (named queen bee acid; C_10_H_18_O_3_; 186.1256), and flavones such as chrysin (C_15_H_10_O_4_; 254.0579) and apigenin (C_15_H_10_O_5_; 270.0528) are influential for wound care, atopic dermatitis, or psoriasis. Also, some compounds such as sterols reduce the water loss on skin wounds and are beneficial for treating atopic dermatitis. The presence of squalene, omega-hydroxy amino acids such as queen bee acid, and specific flavones such as chrysin are responsible for the antiseptic activity. Additionally, it contains *α*-carotene (C_40_H_56_; 536.8726), source of retinol (C_20_H_30_O; 286.2297), which delays collagen degradation and stimulates mitotic division in the epidermis and as a result wound healing (Kurek-Górecka et al., 2020).

#### 3.3.4 Compounds from lard

The *Shiunkō* formula contains a small quantity of lard (1.3% mass). It is therefore only used in high-temperature extraction protocols. However, this ingredient should be taken into account, especially during the extraction process, while changing the fatty acids composition a little. Lard does not contain trans-unsaturated fatty acids but triglycerides with a high rate of saturated fatty acids such as oleic (C_18_H_34_O_2_; 282.2559), palmitic (C_16_H_32_O_2_; 256.2402), stearic (C_18_H_36_O_2_; 284.2715), and linoleic acids (C_18_H_32_O_2_; 280.2402).

Only regarding the therapeutic properties of lard, some studies have investigated the impact of skin applications of animal fat blends on atopic dermatitis lesions. Results showed that the lard mixture significantly ameliorated the macroscopic and microscopic signs and reduced skin thickness and mastocyte incorporation in mice skin lesions. The mixture also had an anti-allergic effect by reducing the involvement of interleukins and E-type immunoglobulins (Lee et al., 2020).

*In vivo,* use of a lard-based ointment on inflamed rat feet reduced paws inflammation. In addition, antioxidant enzymes and inflammatory markers of circulating neutrophils were reduced. A beneficial action of lard is induced by several components such as 5-dodecanolide (C_12_H_22_O_2_; 198.1620), oleamide (C_18_H_35_NO; 281.2719), and resolvin D1 (C_22_H_32_O_5_; 376.2250). This anti-inflammatory effect occurs *via* neutrophils and peripheral mononuclear cells stimulated by lipopolysaccharides, decreasing the production of TNF*α* and the activity of pro-inflammatory enzymes (Capó et al., 2021). These favorable actions suggest that the addition of lard to the *Shiunkō* formula is potentially related to therapeutic activity of interest for skin wound healing and chronic wound treatments. Nevertheless, the low concentration of lard raises questions about its real usefulness as an excipient in the *Shiunkō* formula, especially as it is not present in the other two topical formulas.

### 3.4 Metabolites from Kampo herbal crude drugs involved in wound healing

#### 3.4.1 In *Shiunkō*

##### 3.4.1.1 Purple gromwell root

Purple gromwell root comes from *Lithospermum erythrorhizon* Siebold & Zucc. (Boraginaceae) (The society of Japanese pharmacopeia, 2016). The plant is named *shikon* (シコン; 紫根) or *murasaki* (ムラサキ; 紫) in Japanese. The purple gromwell has long been used as a medicinal plant but also as a dye plant. The pigment produced from the roots is laborious to obtain because of the difficulty of cultivation. It is associated with the most prestigious color considered in Japan. This singular color even indicates the highest grade on “the twelve grades of cap rank,” a classification for court dignitaries established during the Asuka period (592–645).

The cortical part of the roots includes this precious purple pigment with alkannin and shikonin (C_16_H_16_O_5_; 288.0998) which are chiral pairs of hydroxynaphthoquinones. Shikonin has been described as hydroxy-1,4-naphthoquinone (Andújar et al., 2013). With alkannin, both are pigments for food and textiles. These compounds have been well studied for their wound-healing, anti-inflammatory, antimicrobial, antioxidant, antithrombotic, and antitumor properties (Papageorgiou et al., 2008; Andújar et al., 2013). The gromwell pigment comprises acetylshikonin (C_18_H_18_O_6_; 330.1103) and *β*, *ß*-dimethylacrylshikonin (C_21_H_22_O_6_; 370.1416) (Keio University Kampo Department; Cheng et al., 2008). The two shown *in vitro* anti-inflammatory properties resulting from an inhibition of inducible nitric oxide synthase (iNOS) protein expression and a downregulation of nuclear factor kappa-light-chain-enhancer of activated B cells (NF-κB), thanks to the suppression of phosphorylation of extracellular signal-regulated kinases (ERKs) (Cheng et al., 2008).

The root also includes cyanide glycosides such as lithospermoside (C_14_H_19_NO_8_; 329.1111) and polysaccharides. In addition, they promote the migration and proliferation of human keratinocytes and dermal fibroblasts with an increased level of lipid synthesis crucial in the wound healing process (Kim et al., 2012). These metabolites, however, would probably not be in oily extracts because of their hydrophilicity.

Some other compounds from the roots are lithospermic acid (C_27_H_22_O_12_; 538.1111) and caffeic acid (C_9_H_8_O_4_; 180.0423) (Keio University Kampo Department; Thuong et al., 2009). A methanolic extract of the root has shown an effect on serine palmitoyltransferase (SPT) *in vitro* on human keratinocytes (HaCaT cells). This enzyme is crucial for sphingolipid biosynthesis. As a result, purple gromwell root could thus improve the permeability barrier by stimulating the protein level of SPT and could be related to the protective effect on the skin (Thuong et al., 2009). Regarding cosmetical properties, a 5% emulsion containing gromwell root showed moisturizing effects on skin hydration in dose- and time-dependent ways (Chang et al., 2008). Also, the aqueous methanolic extract showed efficiency in the oxidation process because of phenolic and flavonoid compounds. It proves its efficiency *in vitro* against the oxidative stress induced by H_2_O_2_ and ultraviolet radiation on human keratinocytes and neonatal dermal fibroblasts (Yoo et al., 2014). These skin-protective properties suggest that gromwell root is a source of active ingredients for improving wound healing.

##### 3.4.1.2 Pink *Arnebia* root as a substitute of purple gromwell

During meetings with pharmacists specialized in the manufacturing of Kampo medicines in Kanagawa Prefecture, we came to understand that gromwell root could sometimes be replaced by pink *Arnebia* root (*A. euchroma* I.M.Johnst.; Boraginaceae) (アルネビア・エウクロマ). The plant grows naturally in extreme cold and arid environments in the Himalayas, at altitudes of 3,500–4,000 m, and it is an endangered species. The large number of endophytic fungi and bacteria found on the roots and their role in metabolite production suggest the significant variety of metabolites observable (Jain et al., 2021). This crude drug, not listed in the Japanese Pharmacopoeia, has similar attributes to purple gromwell root with the same characteristic purplish color even if they are related to different botanical genera (The society of Japanese pharmacopeia, 2016). *A. euchroma* root is not only visually similar to purple gromwell root but also has many common compounds and therapeutic indications such as wound healing, antimicrobial, and antibacterial properties in Ayurvedic medicine and TCM (Kumar et al., 2021; Ning et al., 2022).

The use of *A. euchroma* root in the preparation of *Shiunkō* ointment will result in a darker-colored formula with the same texture. As far as we know, this variant of the *Shiunkō* formula is not produced with high-temperature extraction protocols and is mostly related to traditional practices rather than large scale production.

In regard to phytochemistry, *A. euchroma* contains coumarins, shikonin derivatives, and also some specific compounds such as arnebinone (C_18_H_22_O_4_; 302.1518), deoxyshikonin (also known as arnebin 7, C_16_H_16_O_4_; 272.1049), and stigmasterol (C_29_H_48_O; 412.3705) (Kumar et al., 2021). About therapeutic properties studied *in vitro*, naphthazarins such as alkannin, shikonin, and their derivatives are strong antibacterial components on methicillin-resistant *S. aureus* and vancomycin-resistant *Enterococci* (Shen et al., 2002). Interesting effects proven on mice have also shown a positive effect of the application of pink *Arnebia* gel (10%) on induced excisional wounds. This study revealed the healing effect of the *A. euchroma* root extract on these wounds and suggested a therapeutic application for chronic wound care (Mohsenikia et al., 2017). The beneficial effect does not only apply to chronic wounds but also burns. An outcome was also evaluated *in vivo* on the mouse model. *A. euchroma* root ointments (with a concentration of 5% or 10%) have shown interesting therapeutic results for burn wound healing compared to 1% silver sulfadiazine topical application (Nasiri et al., 2015). These therapeutic healing and antibacterial properties are of interest to find new possible topical remedies for these diseases and should be further explored.

The fact that some Kampo pharmacists allow themselves to replace purple gromwell root with *A. euchroma* root demonstrates the diversity of Kampo and TCM practices and the interest from a pharmacological point of view. Nonetheless, the geographical origin and scarcity of this product raise concerns of supply and lead us to consider the management of this natural resource in future research.

##### 3.4.1.3 Japanese angelica root

According to the Japanese Pharmacopeia, Japanese angelica is *Angelica acutiloba* (Siebold & Zucc.) Kitag*.* or *A. acutiloba* Kitagawa var. *sugiyamae* Hikino (Apiaceae) (The society of Japanese pharmacopeia, 2016). The second one could nevertheless be considered as a synonym of the first one, but specific literature makes the distinction between the two varieties (WFO, 2022). This has been analyzed according to the sequence of the spacer region between the atpF and atpA genes, which is different among the two varieties (Hosokawa et al., 2006). Also, trnK gene sequences are slightly different for *acutiloba* and *sugiyamae* varieties. The study of trnK and ITS regions in *A. acutiloba* could give information about the origin of Japanese angelica root and describe which variety is closer between *A. acutiloba* var. *acutiloba* and *A. acutiloba* var. *sugiyamae* (Matsubara et al., 2012).

The angelica roots are used as a Kampo crude drug after being passed through hot water (The society of Japanese pharmacopeia, 2016). This ingredient is commonly named *tōki* (トウキ; 当帰), more precisely *Hokkai-tōki* and *Yamato-tōki* for the one historically harvested in Hokkaido and Yamato areas, respectively. The plant is perennial and has been cultivated in Japan and China. The color of the stems from *Yamato*-*tōki* is purplish, while those from *Hokkaido-tōki* are green. The different localities, in addition to the slightly different varieties, reinforce the differences among the metabolites found in *Angelica* roots (Katoh et al., 2011).

Japanese angelica root is traditionally used in Kampo medicine for its immunity activation, analgesic and anti-inflammatory effects. The root contains an essential oil rich in ligustilide (C_12_H_14_O_2_; 190.0994), 3-butylidenephthalide (C_12_H_12_O_2_; 188.0837), and lactone and safrole (C_10_H_10_O_2_; 162.0681) (Keio University Kampo Department). The root has monoterpenoid compounds such as α-pinene or *α*-phellandrene (C_10_H_16_; 136.1252), both phellandrene cyclic monoterpenes with double bond isomers. These two compounds have been studied *in vitro* and *in vivo* on mice and present strong wound-healing activity with better structure and collagen deposition in scar tissue (Salas-Oropeza et al., 2021).

Coumarin derivatives are also in the root such as decursinol angelate (C_19_H_20_O_5_; 328.1311), decursin (C_19_H_20_O_5_; 328.1311), bergapten (C_12_H_8_O_4_; 216.0423), or scopoletin (C_10_H_8_O_4_; 192.0423) (Sowndhararajan et al., 2017). Decursin and decurinol angelate were tested *in vitro* and demonstrated improved wound healing through upregulated transcription of genes encoding extracellular matrix remodeling proteins, inflammatory cytokines, and growth factors in HaCaT human keratinocytes (Han et al., 2018).

The roots include palmitic acid (C_16_H_32_O_2_; 256.2402) and linoleic acid (C_18_H_32_O_2_; 280.2402) which could easily reach the lipophilic phase during the oily extraction process. Polyacetylene compounds such as falcarinol (C_17_H_24_O; 244.1827), falcarindiol (C_17_H_24_O_2_; 260.1776), or falcarinolone (C_17_H_22_O_2_; 258.1620) are also part of the metabolites. Several types of hydrophilic metabolites such as senkyunolide E (C_12_H_12_O_3_; 204.0786), F (C_12_H_14_O_3;_ 206.0943), H (C_12_H_16_O_4_; 224.1049), and I (C_12_H_16_O_4_; 224.1049) and vitamins B_12_ (C_63_H_88_CoN_14_O_14_P; 1354.5674) and B_3_ (C_6_H_5_NO_2;_ 123.0320) are found in the root (Keio University Kampo Department; Jeong et al., 2015). Finally, ferulic acid (C_10_H_10_O_4_; 194.0579), as a derivative of cinnamic acid, is a liposoluble organic acid with antioxidant properties related to the hydroxy and phenoxy groups that can give electrons to quench the free radicals (Srinivasan et al., 2007). Because of its protective role on keratinocytes, fibroblasts, collagen, or elastin, it is a well-known anti-aging compound in the cosmetic industry (Zduńska et al., 2018). Moreover, ferulic acid is particularly beneficial for chronic wounds and diabetic ulcers (Liu et al., 2022). Its effects, whether oral or topical, were tested on streptozotocin-induced diabetic rats using the excision wound model. The treatments strongly influenced the healing process while increasing the epithelialization, inhibiting the lipid peroxidation, and elevating the catalase, superoxide dismutase, glutathione, and nitric oxide (NO) levels (Ghaisas et al., 2014).

Extracts from *Angelica* sp*.* roots are known to induce wound healing, thanks to the modulation of the production of reactive oxygen species (ROS), increased cell migration, fibroblast proliferation, and an inhibition of apoptosis (Hsiao et al., 2012). Lipophilic phthalide derivatives like ligustilide or its enantiomer, Z-ligustilide, are crucial given the *in vitro* studies conducted on human keratinocytes. It suppresses ROS generation through nuclear factor erythroid 2-related factor 2/heme oxygenase-1 (Nrf2/HO-1) upregulation and inflammation by suppressing the NF-κB pathway (Wu et al., 2015).

With regard to the variability and inhomogeneity of metabolites between individuals, the type of treatment carried out on it (such as the temperature and duration of the water boiling treatment on the root) will have an influence. Same goes for the root part used for the extract. Indeed, some metabolites of interest, such as Z-ligustide, will vary according to the type of root part used: high content for the lateral root and low for the root head. Thus, the difference in Z-ligustilide content among Japanese angelica whole root is related to the balance between the root head part and the root other than the head part (Kudo et al., 2021). Consequently, the content of metabolites for wound healing inside the root is sensitive to some factors that are important to consider.

### 3.5 In *Chuōkō*

#### 3.5.1 Turmeric

According to the Japanese Pharmacopeia, turmeric, *ukon* in Japanese (ウコン; 鬱金), is the rhizome of *Curcuma longa* L. (Zingiberaceae) (The society of Japanese pharmacopeia, 2016; WFO, 2022). It can be prepared with or without cork layers, usually with a blanching step (The society of Japanese pharmacopeia, 2016). Originally from India, the plant arrived in Japan a few centuries ago and started to be cultivated in southern prefectures such as Kyushu or Okinawa. Turmeric is the dried rhizome harvested after the flowering period in summer. The rhizome contains not less than 1.0% and not more than 5.0% of total curcuminoids (The society of Japanese pharmacopeia, 2016). This group includes curcumin (C_21_H_20_O_6_; 368.1260), demethoxycurcumin (C_20_H_18_O_5_; 338.1154), and bisdemethoxycurcumin (C_19_H_16_O_4_; 308.1049). Rhizome also contains sesquiterpenes such as caryophyllene (C_15_H_24_; 204.1878), monoterpenes such as eucalyptol (C_10_H_18_O; 154.1358), steroids and lignans (Keio University Kampo Department).

The therapeutic properties of turmeric are well known for topical wound treatments. Curcumin is an asset for remedies containing it, as this molecule acts on the different phases of the wound healing process, from the inflammatory phase to re-epithelialization (Vollono et al., 2019). Studies involving murine models to qualify the wound healing properties were conducted on induced wounds, severe burns, or diabetic ulcers. For burns, a strong influence on the hydroxyproline levels in the skin of the mice treated with turmeric has been proven. Improved collagen deposition, angiogenesis, granulation tissue formation, and epithelialization were demonstrated *in vitro* (Kulac et al., 2013). The same results have been observed on the murine model for induced wounds with a better maturation and cross linking of collagen, increased stability of acid-soluble collagen, aldehyde content, shrinkage temperature, and tensile strength (Panchatcharam et al., 2006). Topical applications of curcumin-enriched remedies also accelerate wound healing in mice by regulating the levels of various cytokines (Yen et al., 2018). Regarding diabetic ulcers, curcumin application increased wound healing and decreased the expression of inflammatory cytokines such as tumor necrosis factor (TNF-*α*), interleukin 1*β* (IL-1*β*), and matrix metallopeptidase 9 (MMP-9). Curcumin also increased the levels of anti-inflammatory modulators such as IL-10, superoxide dismutase, catalase, and glutathione peroxidase. The curcumin application provided granulation tissue improvement with fibroblast proliferation, collagen deposition, and effective epithelialization (Kant et al., 2014). These interesting properties have been the subject of other studies, notably on the effectiveness of gel forms for the topical application of curcumin-based medicines (Mohanty and Sahoo, 2017).

In a nutshell, curcumin acts on the key phases of the healing process such as inflammation (inhibiting the activity of NF- κB, TNF-*α*, and IL-1 cytokines, action on ROS, and a dose-dependent antioxidant effect), proliferation (increased fibroblast migration, granulation, collagen deposition, and re-epithelialization), and remodeling (increased production of transforming growth factor-*β* (TGF) and fibroblast proliferation) (Akbik et al., 2014). All these interesting properties for wound healing need to be considered in Kampo ointments as curcumin is a lipophilic polyphenol substance.

Turmeric also includes terpene-class compounds such as sesquiterpene ketones: ar-turmerone (C_15_H_20_O; 216.1514), *α*-turmerone (C_15_H_22_O; 218.1671), and (+)-*β*-turmerone (C_15_H_22_O; 218.1671). Monoterpenes such as *α*-phellandrene are also found (Avanço et al., 2017). These ketones, as secondary metabolites, are of great interest because of their bacteriostatic and antibacterial properties. Such properties are obviously recognized in the management of chronic wounds where recurrent infections and bacterial resistance are issues. Moreover, a study carried out on turmeric essential oil *in vitro* showed antioxidant, antifungal, and antimycotoxigenic actions on *Fusarium verticillioides* cultures (Avanço et al., 2017). Finally, curcumin has a rather complex biological action on dermatological cell lines with ambivalence regarding apoptosis and wound healing since cell death can be induced while favorable biological effects on wound healing are clearly demonstrated (Mehta et al., 2016). Additionally, studies on the biological effects of Kampo extracts on wound healing should include not only the evaluation of cell proliferation and migration but also the antibacterial component, which is strongly involved in chronic wound management.

#### 3.5.2 Amur cork tree bark

The Amur cork tree, *Phellodendron sp.*, is named *kihada* in Japanese (キハダ; 黄蘗). Its bark, named *ōbaku* (オウバク; 黃柏), is largely used and studied in Asia and is also one of the 50 fundamental herbs known as *huáng bǎi* (黄柏) in Chinese (Ergil et al., 2002). According to the Japanese Pharmacopeia, the bark comes from *Phellodendron amurense* Rupr. or *P. chinense* C.K.Schneid. (Rutaceae), with the periderm removed, revealing a characteristic yellow color (The society of Japanese pharmacopeia, 2016; WFO, 2022). The bark has been traditionally used for its digestive properties for oral intakes and as an anti-inflammatory and antibacterial remedy for topical uses (Li et al., 2006). Amur cork trees are at least 12 years old when the bark is collected during the summer. The inner bark contains at least 1.2% of berberine (C_20_H_18_NO_4_^+^; 336.1236) (The society of Japanese pharmacopeia, 2016). It is a hydrophobic alkaloid isoquinoline of the protoberberine class, a quaternary ammonium salt, and a hydrophilic yellow pigment (Leitao da-Cunha et al., 2005; Patel, 2021).

While berberine is the most distinctive compound, jatrorrhizine (C_20_H_20_NO_4_^+^; 338.1392), palmatine (C_21_H_22_NO_4_^+^; 352.1549), and coptisine (C_19_H_14_NO_4_^+^; 320.0923) are some of the other typical secondary metabolites (Kamimura et al., 2019). The bark contains bitter taste elements such as obacunone (C_26_H_30_O_7_; 454.1991), limonin (also known as obaculactone C_26_H_30_O_8_; 470.1941), and phytosteroids such as *β*-sitosterol (C_29_H_50_O; 414.3862) or campesterol (C_28_H_48_O; 400.3705) (Keio University Kampo Department). In a recent study, the therapeutic action of berberine was evaluated using both *in vitro* and *in vivo* models, first, in a cell model with hyperglycemic treatment and, second, in diabetic rats’ wounds infected *via* streptozotocin induction. These experiments clarified the action of berberine on thioredoxin reductases (TrxR1), a key enzyme in redox homeostasis. This was carried out by suppressing c-Jun N-terminal kinase (JNK) signaling, thereby inhibiting oxidative stress and apoptosis. By taking this action, better cell proliferation was observed. Accelerated wound healing was also correlated with a decrease in the action of MMP-9 and regulation of TGF-β1 and tissue inhibitors of MMP-1 (TIMP1). *In vitro*, treatments with berberine caused an acceleration of wound healing and an improved synthesis of the extracellular matrix. *In vitro*, there was also an inhibition of cell damage induced by hyperglycemic treatments. *In vivo*, for diabetic rats, topical treatment enriched with berberine led to an acceleration of wound healing by stimulation of the TrxR1 enzyme (Zhou et al., 2021). For palmatine, therapeutic actions with the modulation of inflammation through a reduction in the actions of IL-6 and TNF-α have been demonstrated and antibacterial actions on Gram-positive and Gram-negative bacteria. These actions suggest that a beneficial effect could be demonstrated on wound healing cell models (Long et al., 2019).

Regarding metabolites levels of interest in the bark of the Amur cork tree, berberine and palmatine contents differ significantly depending on the botanical origin (*P. chinense* or *P. amurense*), with a lot of berberine and no palmatine for *P. chinense*, lower concentrations of berberine and the presence of palmatine for *P. amurense* (Li et al., 2006). Again, these variabilities suggest that one of the levers for rationalizing Kampo’s use of ointments is a better understanding of the influencing factors on herbal crude drugs’ chemical composition. Here, the botanical species used will be decisive for the composition of therapeutic metabolites in the Kampo ointments.

### 3.6 In *Shinsen taitsukō*

Unlike the two previous formulas containing two herbal crude drugs, the *Shinsen taitsukō* formula contains a mixture of seven herbal crude drugs in equal parts. The presence of *A. acutiloba* root is common with the *Shiunkō* formula. One interesting point is the presence of an herbal crude drug derived from a different species of angelica as the one in *Shiunkō* formula.

#### 3.6.1 Dahurian angelica root

Dahurian angelica root comes from *Angelica dahurica* (Hoffm.) Benth. & Hook. f. ex Franch. & Sav. (Apiaceae) (The society of Japanese pharmacopeia, 2016; WFO, 2022). Dahurica refers to the mountainous region beyond Lake Baikal (Transbaikalia), to the east of it, in Russia. The crude drug is named *byakushi* (ビャクシ; 白芷) in Japanese. Its flavor is characteristic and slightly bitter (The society of Japanese pharmacopeia, 2016). The root was used by Kampo medical doctor Hanaoka Seishū (who created the two remedies *Shiunkō* and *Chuōkō*) for the preparation of anesthetic medication (*Tsūsensan* or *Mafutsu-tō*) used for surgery (Izuo, 2004). Hanaoka Seishū was the first doctor to perform a breast tumor resection under this kind of anesthesia. The use of Dahurian angelica root in this preparation suggests analgesic properties from the crude drug, which is of great interest from an ethnopharmacological perspective.

The root contains more than 300 compounds including 150 coumarins. Among them, there are furanocoumarins such as imperatorin (C_16_H_14_O_4_; 270.0892), byakangelicin (C_17_H_18_O_7_; 334.1052), and oxypeucedanin (C_16_H_14_O_5_; 286.0841). These furanocoumarins are lipophilic compounds and would thus be highly represented in the Kampo oily extracts (Deng et al., 2015). *A. dahurica* root also contains volatile oils (terpenoids, aromatic compounds, and aliphatic compounds) with, for example, *α*-pinene (C_10_H_16_; 136.1252), myrcene (C_10_H_16_; 136.1252), (+)-terpinen-4-ol (C_10_H_18_O; 154.1358), and 1-dodecanol (C_12_H_26_O; 186.1984). In the same way as in the roots of *A. acutiloba*, there is lipophilic ferulic acid (C_10_H_10_O_4_; 194.0579), which is an important phenolic compound. Typical psoralens such as byakangelicol (C_17_H_16_O_6_; 316.0947) compose the root. This kind of metabolites is photosensitizing, and its presence as an ingredient should be monitored (Keio University Kampo Department; Zhao et al., 2022).

Studies carried out on *A. dahurica* root extract for topical uses (also combined to the Chinese rhubarb root extract, another ingredient from *Shinsen taitsukō*) demonstrated significantly wound closure with more inflammatory cell infiltration, collagen fibers, and myofibroblasts a few days after the treatment on rats. It manifested an accelerated wound healing process during the inflammation and proliferation phases (Yang et al., 2017). Other studies made *in vitro* demonstrated an accelerated proliferation of fibroblasts and an upregulated expression of collagen I and III, inducing a better wound healing status (Zhi et al., 2012). *Angelica dahurica* root ethanolic extracts led to an increased proliferation of keratinocytes. Real-time fluorescent quantitative PCR revealed that the cyclin D1 and caspase-3 mRNA levels were downregulated, indicating that apoptosis was inhibited (Bai et al., 2012).

Associated treatment with Chinese rhubarb and *A. dahurica* roots has been studied in more specific chronic wound models with diabetic rats. The mix of herbal extracts improved wound healing in diabetic conditions while increasing vascular endothelial growth factor (VEGF) activities (Chao et al., 2021).

By the way, oral intake of the ethanolic extract from *A. dahurica* roots accelerated diabetic wound healing as well through induced angiogenesis and granulation tissue formation in streptozotocin-induced diabetic rats (Zhang et al., 2017). Angiogenesis is a pivotal process in wound repair, and this therapeutic property of *A. dahurica* root makes its use relevant for wound healing. The oral intake of *A. dahurica* also regulates the polarization of M1 and M2 subtypes of macrophages and thus inflammation (Hu et al., 2021). On the diabetic mice model, oral intake of the *A. dahurica* root extract activated the phosphoinositide 3-kinase/protein kinase B signaling pathway (PI3K/AKT). *In vitro,* the promotion of the hypoxia-inducible factor-1α/platelet derived growth factor *ß* pathway (HIF-1α/PDGF-*β*) efficiently enhanced vascularization in regenerated tissue. This increased neovascularization and PDGF-*β* expression thus facilitated wound healing (Guo et al., 2020).

The benefits brought by *A. dahurica* root oral intake suggest that the topical use may be beneficial, especially its effects on vessel injury-related wounds through the modulation of PDGF-β and VEGF.

#### 3.6.2 Cinnamon bark

Cinnamon bark comes from the trunk of *Cinnamomum cassia* Blume (Lauraceae) with or without the periderm removed (The society of Japanese pharmacopeia, 2016). This species is a synonym of *Cinnamomum cassia* (L.) J. Presl (WFO, 2022). The tree is endemic from Southwest China and North Vietnam, and the bark started to be imported into Japan in the 8th century. Named *keihi* (ケイヒ; 桂皮) in Japanese, it presents a characteristic aromatic, sweet, and pungent taste which is then mucilaginous and slightly astringent (Keio University Kampo Department). Traditionally, the bark is used for its stomachic, hypoglycemic, hypotensive, sedative, and antipyretic activities (Keio University Kampo Department). The bark contains essential oil with aldehydes and esters such as cinnamaldehyde (C_9_H_8_O; 132.0575), 2-methoxy cinnamaldehyde (C_10_H_10_O_2_; 162.0681), and cinnamyl acetate (C_11_H_12_O_2_; 176.0837) as main compounds (Keio University Kampo Department). There are also diterpenoids such as cinncassiol A (C_20_H_30_O_7_; 382.1991), cinncassiol E (C_20_H_30_O_8_; 398.1941), and cinnzeylanol (C_20_H_32_O_7_; 384.2148). The bark contains several sesquiterpenoids such as cinnamosides and sugars. Tannins are found in the bark such as (-)-epicatechin (C_15_H_14_O_6_; 290.0790) or proanthocyanidins such as procyanidin B2 and procyanidin B4 (C_30_H_26_O_12_; 578.1424) (Keio University Kampo Department; Daemi et al., 2019).

The most well-known compound from cinnamon is probably cinnamaldehyde, which has been largely studied *in vitro* and *in vivo* for its wound healing properties. Studies carried out on human umbilical vein endothelial cells (HUVECs) showed that cinnamaldehyde stimulated cell migration and proliferation. It induced a wound healing effect by promoting angiogenesis through activation of the phosphatidylinositol 3-kinase (PI3K) and mitogen-activated protein kinase (MAPK) pathways. Cinnamaldehyde induced *in vivo*, an angiogenic effect on the zebrafish model pre-treated with PTK787 which is a selective inhibitor for VEGFR (Yuan et al., 2018). Cinnamaldehyde as well presented a favorable activity with regards to wound healing in *Pseudomonas aeruginosa*-infected mice. Daily topical application on these induced wounds promoted wound healing and reduced bacteria load. Looking at biochemical parameters, lower IL-17, VEGF, and NO levels have been observed in the cinnamaldehyde-treated wounds (Ferro et al., 2019). In a same way, wound healing activity from the *Cinnamomum* genus has been studied in *C. verum*. Topical application of a hydroethanolic extract enhanced re-epithelialization and keratin biosynthesis in streptozotocin-induced diabetic mice (Daemi et al., 2019).

Epicatechin, a well-known flavonoid from tea leaves, has been tested on radiation-induced cellular damage *in vitro* on fibroblasts and *in vivo* in a zebrafish model. The metabolite increased, *in vitro*, the survival rate and restored the migration ability of the fibroblasts after irradiation. The mechanism is an inhibition of ROS generation, mitochondrial dysfunction, and cell death. Epicatechin reduced the expression of protein kinase p-JNK, p-38 MAPKs, and cleaved caspase-3. In addition, it lowered the cellular damage, improved wound healing after stress such as radiation exposure, and reduced the reprotoxicity in the zebrafish model (Shin et al., 2014). The epicatechin content in cinnamon bark, therefore, suggests that it has important effects on the wound healing process.

#### 3.6.3 *Paeonia lactiflora* root

Peony root from *Shinsen taitsukō* is the root of *Paeonia lactiflora* Pall. (Paeoniaceae) (The society of Japanese pharmacopeia, 2016; WFO, 2022). It contains not less than 2.0% of paeoniflorin (C_23_H_28_O_11_; 480.1632) (The society of Japanese pharmacopeia, 2016). The plant is originally distributed from Northeast China to the eastern part of Siberia, and medicinal hybrids are cultivated in Japan, in Nara and Hokkaido prefectures. *P. lactiflora* root, named *shakuyaku* (シャクヤク; 芍薬), should not be mistaken for the tree peony, *P. suffruticosa* Andrews, whose root cortex is used as a different crude drug. *P. lactiflora* root is traditionally harvested after 5 years of cultivation and then used for its sedative, immunomodulatory, and anti-inflammatory activities (Keio University Kampo Department; He and Dai, 2011).

More distinctive metabolites from the root are monoterpene glycosides such as the isomers, paeoniflorin and albiflorin (C_23_H_28_O_11_; 480.1632), oxypaeoniflorin (C_23_H_28_O_12_; 496.1581), or benzoylpaeoniflorin (C_30_H_32_O_12_; 584.1894). Furthermore, root periderm treatments made on the raw material lead to a loss of bioactive constituents such as paeoniflorin and albiflorin. This has been demonstrated using MALDI MSI studies (Li et al., 2021). Some other important compounds are phenylpropanoids such as paeonol (C_9_H_10_O_3_; 166.06299), hydrolyzable tannins including casuariin (C_34_H_24_O_22_; 784.0759), glycosides such as paeonoside (C_15_H_20_O_8_; 328.1158), and sucrose (C_12_H_22_O_11_; 342.1162) (Keio University Kampo Department; Parker et al., 2016). Some other compounds such as *ß*-glucogallin (C_13_H_16_O_10_; 332.0743), a gallate ester, and benzoic acid (C_7_H_6_O_2_; 122.0368) characterize the *P. lactiflora* root (Sawada et al., 2018). Majority of them are water-soluble sugars, but they will not be included in Kampo oily extracts. Therefore, these compounds mostly represent the singularity of this crude drug. Hence, one of the challenges will be to better understand how oil extraction will influence the composition of the final ointments, especially for sugar-rich crude drugs like this one.

Paeoniflorin has shown effective anti-inflammatory and immunosuppressive activities in a large number of studies. The active mechanisms are associated with regulation of lymphocytes and dendritic cells with an enhancement of several pathways. Thus, protein kinase B (Akt), peroxisome proliferator-activated receptor (PPARγ), protein kinase (PKA), IL-4, IL-10, and TGF-*β* through an inhibition of JNK, ERK, iNOS, cyclooxygenase 2 (COX-2), IL-1*β*, IL-6, IL-17, and interferon *γ* (IFN-*γ*) are intensified by paeoniflorin. Some other signaling pathways such as the G protein-coupled receptor (GPCR), NF-κB, MAPK, and PI3K/Akt are also involved (Zhou et al., 2020). Regarding specific therapeutic actions on chronic wounds, paeoniflorin has been tested on streptozotocin-induced diabetic rat models and high-glucose-treated HaCaT cells. In this study, paeoniflorin improved wound healing in diabetic rat models (diabetic foot ulcer, DFU) and activated the expression of nuclear factor-E2-related factor 2 (Nrf2). *In vitro* experiments showed that paeoniflorin accelerated wound healing through this Nrf2 pathway and an increased expression of VEGF and TGF-β1. It reduced oxidative stress, increased cell proliferation and migration, and decreased apoptosis levels (Sun et al., 2020). A similar study on streptozotocin rat models using skin biopsy punches and high-glucose-treated HaCaT showed a downregulation of IL-*β*, IL-18, and TNF-*α* in paeoniflorin-treated DFU rats. Paeoniflorin decreased the expression levels of the chemokine receptor C-X-C motif chemokine receptor 2 (CXCR2), NF-κB, and p-IκB (Ser36) and increased the IkappaB kinase (IκB) level (Sun et al., 2021).

Paeoniflorin has also been tested with topical applications coupled with hyaluronic acid gel. The metabolite promoted a modulation of macrophages which are well involved in diabetic wounds. The topical application of the paeoniflorin-enriched gel brought better inflammation management, improved angiogenesis, re-epithelialization, and collagen deposition (Yang et al., 2021). Other studies using a paeoniflorin-sodium alginate-gelatin skin scaffold for treating diabetic wounds in a rat model gave positive results on macrophage modulation as well (Yu et al., 2022).

Oxypaeoniflorin, a metabolite found in *P. lactiflora* root, has been studied for its antioxidative and anti-inflammatory activities when it has been associated with paeoniflorin. Observed effects modulated glycation end products and induced inflammatory and oxidative stress responses (Zhanghua et al., 2013). On another note, *ß*-glucogallin reduces the expression of lipopolysaccharide-induced inflammatory markers by inhibiting aldose reductase. Moreover, because *ß*-glucogallin reduces LPS-induced activation of JNK and p38, we could imagine that this kind of plant metabolite may have a positive action on dermatological inflammation (Chang et al., 2013).

All these interesting therapeutic properties for wound healing deserve further research studies, especially a better understanding of what metabolites of interest would pass into the oil fraction during extraction.

#### 3.6.4 *Rehmannia glutinosa* root

*Rehmannia* root is one of the 50 fundamental herbs in TCM, and it is cultivated in the Yamato Valley in Japan and in Northern China (Ergil et al., 2002). According to the Japanese Pharmacopeia, accepted species are *Rehmannia glutinosa* Liboschitz var. *purpurea* Makino and *R. glutinosa* Liboschitz. However, the *purpurea* variety might be considered as a synonym, and nowadays, only the *R. glutinosa* (Gaertn.) DC. (Orobanchaceae) denomination is accepted (The society of Japanese pharmacopeia, 2016; WFO, 2022). The species name comes from the word glutinous because of the sticky aspect of the root (Zhang et al., 2008). Two processes could be used to prepare the crude drug, with the application of steaming (processed: *juku-jiō*) or without it (non-processed: *kan-jiō*) (The society of Japanese pharmacopeia, 2016). The plant is also known as Chinese fox glove and in Japanese *jiō* (ジオウ; 地黄). An interesting fact in relation to *R. glutinosa* is that the plant is sensitive to phytoviruses. Because its reproduction is realized through vegetative propagation, it may facilitate the spread of viral infections (Wu et al., 2002). For Kampo manufacturers, producing virus-free root stocks of *Rehmannia* is therefore important. Regarding pharmacognosy, the virological status of *Rehmannia* plants may be considered an influential factor of metabolites diversity (Matsumoto et al., 1989). The root is traditionally used for its effects on cardiovascular, digestive, and immune systems (Keio University Kampo Department; Zhang et al., 2008).

It contains monoterpenoids, phenethylalcohol glycosides, and triterpenes. Some metabolites are characteristic of the *Rehmannia* botanical genus such as catalpol (C_15_H_22_O_10_; 362.1213) and acteoside (named verbascoside, C_29_H_36_O_15_; 624.2054) (Zhou et al., 2018). These secondary metabolites are sensitive to growing conditions, processing treatments carried out after cultivation, and also the type of virus contamination (Matsumoto et al., 1989; Chang et al., 2006). Among the iridoids, monotermenes and glycosides which distinguish *R. glutinosa* root are the catalpol, dihydrocatalpol (C_15_H_24_O_10_; 364.1369), ajugol (named leonuride, C_15_H_24_O_9_; 348.1420), aucubin (C_15_H_22_O_9_; 346.1264), melittoside (C_21_H_32_O_15_; 524.1741), rehmaglutin A (C_9_H_14_O_5_; 202.0841), rehmaglutin B (C_9_H_13_ClO_5_; 236.0451), rehmaglutin C (C_9_H_12_O_5_; 200.0685), rehmaglutin D (C_9_H_13_ClO_4_; 220.0502), rehmannioside A (C_21_H_32_O_15_; 524.1741), rehmannioside B (C_21_H_32_O_15_; 524.1741), rehmannioside C (C_21_H_34_O_14_; 510.1949), and rehmannioside D (C_27_H_42_O_20_; 686.2269) (Zhang et al., 2008). Moreover, the root is rich in saccharides with three types of monosaccharides extracted (glucose, galactose, and fructose) and five kinds of oligosaccharides. Amino acids like arginine (C_6_H_14_N_4_O_2_; 174.1117) and organic acids like benzoic, linoleic, and stearic acids are also a part of the root (Keio University Kampo Department). In a way, the compounds present in *Rehmannia* root are either hydrophilic with the wide variety of compounds such as glycosides or more lipophilic with the organic acids.

Iridoids are a metabolites group characteristic of the *Rehmannia* botanical genus and the *Rubiaceae* and *Scrophulariaceae* botanical families. This group presents anti-inflammatory activity that may be beneficial in the treatment of inflammation (Viljoen et al., 2012). Moreover, the wound-healing activity of acylated iridoid glycosides was shown *in vitro* to stimulate the growth of human dermal fibroblasts (Nishimura et al., 1989; Stevenson et al., 2002). Amino acids in the root, even at low concentration, could therefore have a positive action on wound strength and collagen deposition. These therapeutic effects brought about by oral supplementation have been tested in artificial incisional wounds in animal models (Stechmiller et al., 2005).

In the same way, as discussed earlier for the compounds present in sesame oil, *R. glutinosa* root is rich in omega 6. It has favorable properties such as modulation of cell migration and proliferation, phagocytic capacity, and the production of inflammatory mediators (Silva et al., 2018). The underground parts of *Rehmannia* therefore include both lipophilic and hydrophilic compounds (with the iridoids in particular). Further research may reveal the metabolites present in Kampo oil extracts and thus provide a better understanding of the therapeutic actions of Kampo ointments.

#### 3.6.5 Chinese rhubarb root

Chinese rhubarb root usually comes from species *Rheum palmatum* L., *R. tanguticum* Maxim. ex Balf., *R. officinale* Baill., or *R. coreanum* Nakai (Polygonaceae) (The society of Japanese pharmacopeia, 2016; WFO, 2022). The plant also spreads to Eastern Europe, Northern America, and the cold regions of Asia. Since ancient times, it has been used as a remedy in these areas. The plant is named *daiō* (ダイオウ; 学名) in Japanese. Roots are usually used after a few years of cultivation. The interspecific hybrids made from *Rheum coreanum* Nakai x *R. palmatum*, named *shinsyu-daiō*, are now largely cultivated in Japan.

Chinese rhubarb root is one of the most commonly used Kampo ingredients, traditionally chosen for its laxative and tonic properties (Keio University Kampo Department). The crude drug presents antibacterial, digestive, anti-inflammatory, anti-fibrosis, and antitumor activities (Xiang et al., 2020).

At least 30 compounds could be identified in the Chinese rhubarb root. From anthraquinones and associated glucosides, dianthrones, phenylbutanones, stilbenes, flavan-3-ols, procyanidins, glucogallin, acyl-glucoses, gallic acid, or polymeric procyanidins were identified. Major differences regarding compounds concentrations exist depending on the botanical and geographical origins of the plant (Komatsu et al., 2006). According to the Japanese Pharmacopeia, the Chinese rhubarb root contains not less than 0.25% of sennoside A (C_42_H_38_O_20_; 862.1956) (The society of Japanese pharmacopeia, 2016). This sennoside is from the anthraquinones group, but some other representatives are part of the root such as emodin (C_15_H_10_O_5_; 270.0528), rhein (C_15_H_8_O_6_; 284.0321), aloe-emodin (C_15_H_10_O_5_; 270.0528), chrysophanol (C_15_H_10_O_4_; 254.0579), or physcion (C_16_H_12_O_5_; 284.0685). The root contains diantrone compounds such as sennoside F (C_44_H_38_O_23_; 934.1804) or sennidin A (C_30_H_18_O_10_; 538.0900) (Koyama et al., 2007). Other metabolites, such as tannins or other glycosides, are also typical, but they will not be easily extracted with sesame oil. The tannin group from Chinese rhubarb includes (+)-catechin and (−)-epicatechin (C_15_H_14_O_6_; 290.0790). Both of these enantiomeric compounds are antioxidant flavonoids. Furthermore, some other glycosides such as lindleyin (C_23_H_26_O_11_; 478.1475), trans-stilbene (C_14_H_12_; 180.0939), naphthalene (C_10_H_8_; 128.0626), and phenylbutanone are representatives of Chinese rhubarb root (Keio University Kampo Department). An important compound from the root is gallic acid, a trihydroxybenzoic acid (C_7_H_6_O_5_; 170.0215).

Anthraquinones have broad biological activities that do not concern therapeutic applications for the digestive sphere. Indeed, emodin, a trihydroxyanthraquinone, has shown *in vitro* action on the NF-κB and phosphoinositide 3-kinase/Akt pathways, which are well implicated in the cell cycle. *In vitro* and *in vivo* studies have shown an anti-fibrosis action at the skin level *via* treatments of hypertrophic scars involving fibroblast deregulation (Mehta et al., 2016). This was confirmed in another study conducted on rats with topically applied emodin that enhanced the repair of excisional wounds. In this study, tissue regeneration stimulation implicated the SMAD-mediated TGF-β signaling pathway (Tang et al., 2007). According to recent *in vitro* studies, some other anthraquinone metabolites such as rhein stimulate HaCaT cell proliferation through the activation of the estrogen signaling pathway. This induces the expression of proto-oncogene c-myc in collaboration with the protein FosB and proto-oncogene JunD. Together, these activations accelerate re-epithelialization, an important step in the wound healing process (Xu et al., 2022).

In addition to this, gallic acid showed a beneficial role in wound healing. Thanks to the regulation and activation of the wound healing mechanisms surrounding the fibroblasts which are observed *in vivo* after *per os* treatment in experimentally induced hyperglycemic animals. The favorable factors observed included better wound edge cohesion, a smaller wound area, and a shorter time to reach re-epithelialization after wound induction (Liu et al., 2022).

The fact that Chinese rhubarb contains such compounds favorable for wound healing deserves metabolomics analyses of these compounds which are lipophilic (rhein or gallic acid) or amphiphilic (emodin) to understand their distribution in Kampo oily extracts.

#### 3.6.6 *Scrophularia* root

The genus *Scrophularia* gathers more than 100 species of herbaceous flowering plants commonly known as figwort (Scrophulariaceae). There are two main species used in TCM and Kampo. First one is the *Scrophularia ningpoensis* Hemsl, with a species’ name which is refering to Ningbo, a city from Zhejiang province in China, also named *Ningpo* in the past*.* The second is *S. buergeriana* Miq. (WFO, 2022). Both species are named *genjin* (ゲンジン; 玄参) in Japanese. The root part is essential in traditional medicine from East Asia and has been used there for 2,000 years (Zhang et al., 2021). According to the Chinese Pharmacopoeia, *Scrophularia* roots are used to treat inflammatory and infectious pathologies. The crude drug is however not referenced by the Japanese Pharmacopoeia (The society of Japanese pharmacopeia, 2016). The name of the genus *Scrophularia* is also related to its topical use on scrofula lesions (tuberculous cervical lymphadenitis) (Lu et al., 2019). This suggests its properties as a relevant ingredient for skin wound healing treatments and as herbal antiseptic. Moreover, both *S. ningpoensis* and *S. buergeriana* have shown anti-inflammatory effects in *in vitro* and *in vivo* studies (Lee et al., 2021).

The *Scrophularia* botanical genus is a rich source of iridoids, terpenes, phenolic glycosides, alkaloids, and flavonoids (Pasdaran and Hamedi, 2017; Zhang et al., 2021). These compounds play a role as antioxidants, as proven *in vivo* through metabolomic approaches (Lu et al., 2019). Therapeutic applications of *S. ningpoensis* root, in addition to those for infectious lesions, have shown an effect on skin inflammations caused by allergic reactions in mice (Zhang et al., 2021). The anti-inflammatory properties studied in aqueous extracts affected the MAPK pathway and inhibited the NF-κB pathway (Shen et al., 2012). However, these are not easily extrapolated to Kampo extracts made with sesame oil.

Another characteristic compound is 6’-*O*-cinnamoylharpagide (C_24_H_30_O_11_; 494.1788), which has been isolated from the ethanolic extracts made using the roots of *S. ningpoensis*. It is accompanied by other metabolites such as harpagide (C_15_H_24_O_10_; 364.1369), harpagoside (C_24_H_30_O_11_; 494.1788), 8-*O*-feruloylharpagide (C_25_H_32_O_13_; 540.1843), 8-*p*-coumaroylharpagide (C_24_H_30_O_12_; 510.1738), 6-*O*-methylcatalpol (C_16_H_24_O_10_; *376.1369),* aucubin (C_15_H_22_O_9_; 346.1264), buergerinin B (C_9_H_14_O_5_; 202.0841), teuhircoside (C_15_H_20_O_9_; 344.1107), and 6-*O*-cinnamoyl-*d*-glucopyranose (C_29_H_26_O_15_; 310.1052) (Niu et al., 2009). The properties of acylated iridoid glycosides from *S. nodosa*, a close species, were studied *in vitro* and showed a beneficial, dose-dependent effect on human fibroblasts (Stevenson et al., 2002).

The history of herbal medicines’ uses from the genus *Scrophularia* and its specific metabolites suggest that the root plays an important role in wound healing and the management of dermatological superinfections.

## 4 Discussion

To our knowledge, there is no literature describing the composition and mode of action of Kampo ointments extracted *via* oily base. Only a few clinical trials have been conducted on these specific traditional remedies (Huang et al., 2004; Lu et al., 2008; Chak et al., 2013; Nakase et al., 2019). It can be assumed that the reason for this is certainly due to the experimental difficulties encountered both in biological testing and in metabolomic analysis. Testing oily based herb extracts represents practical challenges of solubility and homogeneity related to chemical and biological experiments. For chemical analysis, the oily and waxy nature of Kampo ointments poses a problem of solubility within the analytical solvents used in liquid chromatography. In addition, the fouling of the source in liquid chromatography coupled to mass spectrometry induces disturbances in the collected signal. These problems lead to more complex metabolomic analyses. Concerning biological analyses aiming at evaluating the therapeutic action of Kampo products on cellular models, other obstacles are encountered. The solvents usually used such as ethanol, non-ionic surfactants (polysorbates 20 or 80), or dimethyl sulfoxide (DMSO) do not allow effective suspension of the compounds and generate toxicity in the cell types tested.

Nonetheless, if the biomechanisms of the Kampo ointments are not yet elucidated possibly because of these challenging analysis difficulties, the metabolites present in the crude drugs have already been evaluated for dermatological applications. This is the case for oleic acid which is known to be anti-inflammatory, and the fact that Western medicines generally use fatty topical products for their protective effects (Lin et al., 2017). This supports the interest in using such lipophilic ointments. In the same way, some plants found in Kampo ointments, such as figwort (genus *Scrophularia*), are also used in other folk and traditional remedies for the treatment of skin pathologies (Pasdaran and Hamedi, 2017). This convergence of knowledge suggests that there is a lot to discover about the therapeutic modes of action involving Kampo ointments and wound healing.

During this bibliographic work, we found a lot of information on the herbal medicines included in the three Kampo remedies, but we came up against two obstacles. First, many of the publications considered different routes of administration than the topical one. Also, for some of the topical applications of herbal extracts evaluated, the type of extraction was different from the characteristic oily extraction used for Kampo ointments. The remedies we are studying are indeed used dermally and are derived from herbal extracts produced in an oily medium. Therefore, much of the information from the literature is not directly applicable. Second, very few publications or sources mention Kampo directly, whereas we found more information for other traditional medicines such as TCM.

This review gathers information on three ointments that traditional Japanese Kampo uses, namely, *Shiunkō*, *Chuōkō*, and *Shinsen taitsukō*. Although the biological activities of commercial Kampo extracts are not known, tradition has validated them. This is nuanced since ethnopharmacology refers to some traditional uses that persist independently of a proven therapeutic effect.

Considering the herbal medicines involved in Kampo ointments, several important secondary metabolites are lipophilic products such as curcumin (*Curcuma longa*), shikonin (*Lithospermum erythrorhizon*), furanocoumarins such as imperatonin and byakangelicin (*Angelica acutiloba*), and anthraquinones such as rhein or gallic acid (*Rheum palmatum*). If the chemical composition of Kampo oily extracts has not yet been described to our knowledge, there is good reason to believe that the molecules described in this review may be present.

With regard to the rationalization of the use of Kampo ointments, there is much to be carried out in terms of understanding the biotic and abiotic factors influencing their chemistry. Indeed, botanical species and varieties (for the genera *Angelica, Phellodendron, Rheum,* or *Scrophularia*), growing conditions, type of crude drugs used, and processing types are important vectors of metabolites variability among herb batches. Therefore, a better understanding of the factors influencing the levels of metabolites of interest in medicinal plants and hence in crude drugs and ointments is needed.

These natural therapeutics are based on tradition, and *in vitro* and then *in vivo* evidences would be crucial arguments for a possible rationalization of these traditional remedies. In the case of Kampo ointments for dermatological uses, an additional constraint comes from the herbs extraction type in oil. This requires adaptations by biologists and chemists to achieve a good understanding of these unique remedies. Adapted metabolomic approaches and specific biological assays should allow us to learn more about the Kampo ointments used for skin wound healing.
